# Novel Trypanocidal Inhibitors that Block Glycosome Biogenesis by Targeting PEX3–PEX19 Interaction

**DOI:** 10.3389/fcell.2021.737159

**Published:** 2021-12-20

**Authors:** Mengqiao Li, Stefan Gaussmann, Bettina Tippler, Julia Ott, Grzegorz M Popowicz, Wolfgang Schliebs, Michael Sattler, Ralf Erdmann, Vishal C Kalel

**Affiliations:** ^1^ Department of Systems Biochemistry, Faculty of Medicine, Institute of Biochemistry and Pathobiochemistry, Ruhr University Bochum, Bochum, Germany; ^2^ Institute of Structural Biology, Helmholtz Zentrum München, Neuherberg, Germany; ^3^ Department of Chemistry, Bavarian NMR Center, Technical University of Munich, Garching, Germany

**Keywords:** neglected tropical diseases, trypanosoma, glycosome biogenesis, protein–protein interaction, PPI inhibitors, alphascreen, small-molecule inhibitor screen, PEX3-PEX19 inhibitor

## Abstract

Human pathogenic trypanosomatid parasites harbor a unique form of peroxisomes termed glycosomes that are essential for parasite viability. We and others previously identified and characterized the essential *Trypanosoma brucei* ortholog TbPEX3, which is the membrane-docking factor for the cytosolic receptor PEX19 bound to the glycosomal membrane proteins. Knockdown of TbPEX3 expression leads to mislocalization of glycosomal membrane and matrix proteins, and subsequent cell death. As an early step in glycosome biogenesis, the PEX3–PEX19 interaction is an attractive drug target. We established a high-throughput assay for TbPEX3–TbPEX19 interaction and screened a compound library for small-molecule inhibitors. Hits from the screen were further validated using an *in vitro* ELISA assay. We identified three compounds, which exhibit significant trypanocidal activity but show no apparent toxicity to human cells. Furthermore, we show that these compounds lead to mislocalization of glycosomal proteins, which is toxic to the trypanosomes. Moreover, NMR-based experiments indicate that the inhibitors bind to PEX3. The inhibitors interfering with glycosomal biogenesis by targeting the TbPEX3–TbPEX19 interaction serve as starting points for further optimization and anti-trypanosomal drug development.

## Introduction

Trypanosomatids are vector-borne protozoan parasites responsible for highly divergent range of eukaryotic infections in humans and animals. Particularly in the tropical and sub-tropical regions of the world, *Trypanosoma brucei* (*T. brucei*), *T. cruzi*, and various *Leishmania* species cause African and American trypanosomiases and leishmaniasis, respectively. *T. brucei* sub-species cause human infections termed African sleeping sickness (human African trypanosomiasis, HAT), and its close related species *T. congolense* and *T. vivax* cause animal infections termed nagana disease in sub-Saharan regions. The human infections are fatal without treatment and affect across 36 countries in sub-Sahara African area, and majority of the reported cases (>95%) were caused by the sub-species *T. brucei gambiense* (Kennedy, 2019; WHO). In addition, nagana has been a burden for economic development by affecting domestic animals (Richards et al., 2021). More than 20 million people are currently infected with *T. cruzi* or *Leishmania*, leading to over 30 thousand deaths each year. With the fact that there is no effective vaccine against HAT due to the antigenic variation, chemotherapies have been the only major approach for treating the diseases. The well-known frontline drugs, suramin, pentamidine, melarsoprol, and eflornithine have various limitations, i.e., they are constrained by stage and causative-strain of the disease, toxicity, logistical issues, and the emergence of drug resistance. Furthermore, the fifth drug nifurtimox has been used off-label in the combination therapy with eflornithine (NECT) to treat second-stage *T. b. gambiense* infections. Melarsoprol remains the only treatment for stage 2 infection caused by *T. b. rhodesiense* (Pépin and Milord, 1994; Wang, 1995; Babokhov et al., 2013; Büscher et al., 2017).

Despite a long history for treatments with these compounds, the cellular targets were not clear for a long time. Except for eflornithine, which is an irreversible inhibitor of ornithine decarboxylase that functions in the spermidine biosynthesis. Extensive studies have been performed to identify the mode of actions of these drugs. To be specific, it has been reported that melarsoprol and suramin target mitosis and cytokinesis, respectively. In addition, nifurtimox and pentamidine interfere with the parasite mitochondria by disrupting its membrane potential and inducing loss of kinetoplast DNA (Alsford et al., 2012; Thomas et al., 2018). Inhibitors of known targets in rational drug development have been reported in the past decades, including phenothiazine that blocks trypanothione reductase (Chan et al., 1998; Khan et al., 2000; Persch et al., 2014); other targets are purine metabolism of parasites (El Kouni, 2003), *T. brucei* topoisomerase IB (Bakshi and Shapiro, 2004), glycosomal enzymes glycerol kinase (Balogun et al., 2019), and phosphofructokinase (McNae et al., 2021), as well as proteins involved in the glycosome biogenesis (Dawidowski et al., 2017; Banerjee et al., 2019; Kalel et al., 2019).

Fexinidazole has been the first oral treatment for HAT, and its treatment for both stages of *T. b. gambiense* HAT was approved by the European Medicines Agency’s (EMA) Committee in 2018. In 2019, the compound was added to the WHO Essential Medicines List and very recently was approved by the United States Food and Drug Administration (FDA) (Mullard, 2021). Using of the compound with rhodesiense HAT is still undergoing clinical trial (DNDi). Fexinidazole is a prodrug activated by NADH-specific nitroreductase (NTR1), and the resulting highly reactive nitro-reduced products kill parasites by hitting multiple targets. Fexinidazole is also interested for potentially targeting *T. cruzi*, which is the causative agent for the Chagas disease; *T. cruzi* harbors an orthologous nitroreductase enzyme (Dickie et al., 2020). The cellular target of acoziborole and the related benzoxaborole AN7973 is RNA cleavage and polyadenylation specificity factor subunit (CPSF3) (Begolo et al., 2018). To be specific, clinical trials with acoziborole, as another oral treatment, have been completed in 2020. The drug is now undergoing approval by EMA and FDA (DNDi). Because of decades of efforts in research and control of the HAT, a number of recorded new cases were decreased to 992 in 2019 (WHO). *T. brucei*, however, remains as the very important model organism for studies in resolving potential cellular targets for the closely related parasites. It is experimentally more amenable model system compared to *T. cruzi* and *Leishmania* species, responsible for the infections of higher impacts, which demand updating in therapeutic strategies. Moreover, *T. brucei* has been verified as a valid model system for *T. cruzi*, for compounds targeting the PEX14 and PEX5 interaction (Dawidowski et al., 2017). Last but not the least, animal infections of livestock (nagana) caused by *T. congolense*, *T. vivax*, and *T. brucei* species, leading to the annual loss of over 4 billion United States dollars are remaining great challenges (Shereni et al., 2021). This demonstrates the importance of using *T. brucei* as the model organism for drug development.

Trypanosomatid parasites harbor a unique form of peroxisome termed glycosome, which compartmentalizes the first seven enzymes of the glycolytic pathway (Opperdoes and Borst, 1977). Unlike peroxisomes, glycosomes are essential for the survival of bloodstream form (BSF) parasites as glycolysis is the sole source of ATP in this stage. Defects in the glycosome biogenesis lead to mislocalization of glycolytic enzymes to the cytosol, where their unregulated enzyme activities deplete cellular ATP levels and accumulate glucose metabolites to the toxic levels that kills the BSF parasites (Bakker et al., 1999; Furuya et al., 2002; Haanstra et al., 2016). Glycosomal matrix and membrane protein import involves distinct sets of Peroxin (PEX) proteins. Small-molecule inhibitors of the TbPEX14–TbPEX5 interaction that block the glycosomal matrix protein import are lethal to the *Trypanosoma* parasites (Dawidowski et al., 2017) and have recently established the import machinery and glycosome biogenesis as novel therapeutic targets for the development of trypanocidal drugs. Peroxisomal membrane protein (PMP) import is mediated by PEX19, PEX3, and PEX16 (Giannopoulou et al., 2016). PEX19 is the cytosolic receptor and chaperone for newly synthesized PMPs, which targets the cargo PMPs to the peroxisomal membrane by docking at PEX3 (Fang et al., 2004; Jones et al., 2004). Mammalian PEX16 and its functional homolog Pex36 in yeast are involved in ER-to-peroxisome trafficking of PMPs (Honsho et al., 2002; Farré et al., 2017). TbPEX19 and TbPEX16 have been identified and characterized previously (Banerjee et al., 2005; Kalel et al., 2015). We and others recently identified a highly divergent *Trypanosoma* ortholog of PEX3 with very low sequence identity with the known PEX3 proteins from other organisms (Banerjee et al., 2019; Kalel et al., 2019). TbPEX3 was shown to be essential for the parasite survival, because RNA interference (RNAi) knockdown of TbPEX3 expression is lethal to the trypanosomes.

The TbPEX3–TbPEX19 interaction is expected to be an attractive candidate drug target because 1) TbPEX3 acts in the early stages of glycosome biogenesis, particularly in the recruitment of PMPs to the glycosome, which subsequently affects matrix protein import. 2) Sequence similarity of TbPEX3 to its human homolog is low. Here, we report the development of a high-throughput assay to screen small-molecule inhibitors of the TbPEX3–TbPEX19 interaction. We have identified compounds that act on-target in trypanosomes to disrupt glycosome biogenesis and kill *Trypanosoma* parasites but with no apparent toxicity to mammalian cells. The establishment of the high-throughput assay and novel TbPEX3–TbPEX19 inhibitors serve as the starting points for further optimization to develop novel therapies against trypanosomatid parasite infections.

## Methods

### Molecular Cloning

The *TbPEX3* gene (Tb427tmp.01.2020) was amplified from genomic DNA, to tag the soluble fraction of TbPEX3 (residues 45–476) with N-terminal GST tag, the *TbPEX3* gene was amplified with primers RE6944 and RE6945 spanned by *Bam*HI and *Sal*I sites and cloned into vector pGEX-4T2. The full-length *TbPEX19* gene (Tb427tmp.211.3300) was amplified using primers RE7130 and RE7033 containing *Nco*I and *Xho*I sites, by cloning of the gene into vector pET24d; the expressed protein was tagged C-terminally by six Histidine residues. *TbPEX19* was cloned downstream of a His_6_ tag and a TEV cleavage site, into the vector pCDF using the method of SLIM (site directed, ligase-independent mutagenesis), with primers: TbPEX19F, TbPEX19R, pCDF11F, and pCDF11R. *TbPEX19* was cloned into pCOLA with C-terminal tagging of StrepII (RE7146 and RE7148). The *TbPEX11* gene (Tb427tmp.01.3370) was cloned into vector pGN1 using *Bst*BI and *Bam*HI sites (primers RE8070 and RE8071) with GFP tag at its C-terminus. Primers are shown in Table 1.

**TABLE 1:** Primer list.

Primer name	Sequence (5′-3′)
RE6944	CGG​GAT​CCC​CCG​TGC​AAA​ACA​GCA​TTG​TTG
RE6945	ACG​CGT​CGA​CTT​ATA​AAT​CGC​GGC​ATG​TAA​CTC​TAA​TCG​TCT​C
RE7033	CCG​CTC​GAG​CAC​TGA​TGG​TTG​CAC​ATC​GGC​AAG​TC
RE7130	CAT​GCC​ATG​GAT​GTC​TCA​TCC​CGA​CAA​TGA​CGC​CG
RE7131	GAA​TTC​TCA​TGC​ACT​CTT​CTC​GAA​TTG​TGG​GTG​AGA​CCA​CAC​TGA​TGG​TTG​CAC​ATC​GGC​AAG​TC
RE7148	CAT​GCC​ATG​GGC​ATG​TCT​CAT​CCC​GAC​AAT​GAC​GCC​G
RE8070	AAG​AAT​TCG​AAA​TGT​CTG​AGT​TCC​AAA​GGT​TTG​TT
RE8071	AAG​ACG​GAT​CCG​ATT​TGA​TCT​TGT​TCC​AGT​TCA​A
TbPEX19F	ATG​TCT​CAT​CCC​GAC​AAT​GAC​G
TbPEX19R	TTA​CAC​TGA​TGG​TTG​CAC​ATC​GGC
pCDF11F	GAA​TCT​TTA​TTT​TCA​GGG​CAT​GTC​TCA​TCC​CGA​CAA​TGA​CG
pCDF11R	CGC​CTT​GTG​ACG​TGT​CTT​ACA​CTG​ATG​GTT​GCA​CAT​CGG​C

### Recombinant Protein Overexpression and Purification

*E. coli* BL21(DE3) cells were transformed with corresponding plasmids, and protein expression was induced when OD_600_ reached ∼0.6. Protein expression of either GST-TbPEX3d44 alone or dual expression with TbPEX19-His was initiated by addition of 0.4 mM IPTG, followed by growth for 16 h at 18°C. The expression of TbPEX19-His and GST-His (pET42b) was induced with 1 mM IPTG, and cells were cultured for 3 h to allow overexpression. The *E. coli* BL21(DE3) cultures were harvested, and clarified supernatant was prepared as described in Kalel et al. (2019). GST-TbPEX3d44 and the co-expressed complex were captured with affinity chromatography using glutathione agarose beads (Protino^®^, Macherey-Nagel). TbPEX19-His was purified by Nickel-NTA resin (Protino^®^, Macherey-Nagel) using gravity-flow columns (30-μm pore size, Pierce^®^). Protein-bound beads were washed with 5× volume of phosphate-buffered saline (PBS) (pH 7.4), and proteins were eluted with either 10 mM reduced glutathione or 200-mM imidazole supplemented with PBS buffer. To produce tag-free TbPEX19, His-TbPEX19 was purified similarly and incubated with His-tagged TEV protease, and the cleaved-off His tag and TEV protease were removed by Ni^+^-NTA resin. TbPEX19-Strep was purified using StrepTactin Sepharose resin according to the user manual (IBA). Purified proteins were loaded into size-exclusion chromatography column (Superdex^®^ 200 10/300 GL), and the predicted size of the co-migrated complex (GST-TbPEX3d44 and TbPEX19-His) was around 117 kDa by comparing with the calibration curve using the same column (data not shown). Protein aliquots were snap-frozen with liquid nitrogen and stored at −80°C.

### High-Throughput Compound Screening

Co-expressed TbPEX3–TbPEX19 (5 nM) and GST-His (54 nM) were used for the primary and counter screens, and these protein concentrations provided strong and very similar range of signal window to allow reliable statistical analysis (data not shown). Screening of 4,480 diversity-oriented compounds (DIVERSet-CL, collection No. 1511-1, ChemBridge) was performed in the format of 384-well plates (AlphaScreen-384 plates, PerkinElmer^®^). The 25-μl reactions consist of 10 μl of protein solution (5 nM for PEX3/PEX19 complex and 54 nM for GST-His; all concentrations for the Alpha assays were final concentrations unless otherwise stated), 5 μl of compound solution (10 μM), and 5 μl of solution for each of the donor and acceptor beads (1:1,250, v/v). The above solutions were prepared in the reaction buffer [0.5% BSA v/v, 0.05% Tween 80 v/v, 0.2 mM DTT, PBS (pH7.4)] on the day of assay, diluting the compounds from 1 mM stocks in DMSO. Compounds were incubated with the proteins for 30 min at room temperature (RT). Five microliters of AlphaScreen Nickel-chelate acceptor beads (cat. no. 6760619C, PerkinElmer^®^) and AlphaScreen Glutathione donor beads (cat. no. 6765300, PerkinElmer^®^) were distributed to the mixture consecutively, with a 15-min interval. The complete 25-μl reaction solutions were incubated for 45 min at RT in the dark, and Alpha signals were captured with Cytation 5 plate reader (BioTek^®^) with the gain value set at 180. Schematic representation of the experiment setup of the high-throughput screening assays was prepared with BioRender.com.

### Estimation of the Binding Affinity of the TbPEX3–PEX19 Interaction Using AlphaScreen Approach

Binding affinity of TbPEX3 and TbPEX19 was estimated in the formats of 1) saturation binding assays and 2) competitive binding assays; each of the assays was performed with triplicates, and drug candidates were substituted with buffer. 1) Constant concentrations of TbPEX19-His (0.3, 1, and 10 nM) were saturated with serial dilutions of GST-TbPEX3 from 0 to 300 nM. The saturation curves were fitted with the one-site specific binding model with GraphPad Prism 9; the mean of apparent equilibrium dissociation constants (*K*_*D*_) from the best fits was obtained from three independent assays with varied concentration of TbPEX19-His. 2) TbPEX19-His (10× or 5×) was used to saturate GST-TbPEX3d44 (0.2, 0.3, 1, and 2 nM). Serial dilutions of either tag-free TbPEX19 (0–5 μM) or TbPEX19-Strep (0–7.2 μM) were used to compete away the TbPEX19-His from its complex with GST-TbPEX3d44. Alpha signals were normalized to % for comparison between assays, and curves were fitted using one-site homologous model in GraphPad, which assume that tag-free TbPEX19 and TbPEX19-Strep binds in identical way as of TbPEX19-His to GST-TbPEX3d44.

### Hit Selection From the Screen

Individual assays with the Z’ factor above 0.5 (Zhang et al., 1999) indicate good assay capacity in distinguishing between positive and negative controls. Hits were selected by the criteria 1) 50% signal cutoff and 2) robust Z-score ( ≤ 3) (Malo et al., 2006; Chung et al., 2008; Birmingham et al., 2009). Following initial hit selection, 10 μM of drugs were tested with GST-His (54 nM) and TbPEX19/TbPEX3 (5 nM) in smaller scale, and the level of signal was normalized (in %) and compared. Compounds with specific inhibition activity were prioritized. IC50 of four candidate compounds targeting the interaction of TbPEX3–TbPEX19 (compound **1**, **2**, **3**, and **4**) were tested by incubation of the complex with serial dilutions of compounds from 0 to 100 μM.

### ELISA Assays

Dose-dependent responses of the inhibitors were analyzed with TbPEX19–TbPEX3 interaction. One hundred microliters of TbPEX19-His (10 μg/ml) diluted in PBS (pH 7.4) was coated on 96-well plates (Immulon^®^ 2 HB, Thermo Fisher Scientific) at RT for 1 h. Wells were washed twice with 250 µl of PBS to remove unbound protein and blocked with 200 µl of buffer C [3% BSA in PBS (pH 7.4)] for 1 h. The inhibitors were diluted to desired concentrations in PBS, and 100 µl of each of the compounds were added to TbPEX19-coated wells, followed by 1-h incubation. To these wells, 100 µl of GST-TbPEX3d44 was added to reach final concentration of 0.3 nM and incubated for 1 h further. After three washes with PBS, bound GST-TbPEX3d44 was detected by mouse monoclonal anti-GST antibody (Sigma-Aldrich, 1:1,000 v/v in buffer D) [0.05% v/v Tween 20 in PBS (pH 7.4)]; signal was amplified by rabbit anti-mouse horseradish peroxidase (1:1,000 v/v in buffer D, Invitrogen). Substrate 3,3′,5,5′-tetramethylbenzidine (TMB, Thermo Fisher Scientific) was added to initiate the colorimetric reaction, which was terminated after 20 min by adding H_2_SO_4_, and the absorbance was measured at wavelength of 450 nm.

ELISA with TbPEX14–TbPEX5 was used as an independent assay to confirm compound specificity by examining the compound activity on TbPEX14–TbPEX5 interaction. TbPEX14–TbPEX5 assays were performed similarly with following changes. GST-TbPEX14_1-84_ was coated, and biotinylated TbPEX5 peptide (Biotin-Aca-Aca-EQWAQEYAQMQAM) was used as analyte to a final concentration of 500 nM. Bound PEX5 was detected using streptavidin-conjugated alkaline phosphatase [1:2,000 v/v in PBS, 0.05% v/v Tween 20 (pH 7.4), buffer D, Promega] and *p*-nitrophenylphosphate (PNPP, Thermo Fisher Scientific) as a substrate, reactions stopped with 3 M NaOH and absorbance read at 405 nm.

### *Trypanosoma* Culture, Transfection and Cell Viability Assays

BSF strain Lister 427 (termed hereafter as BSF427) and cell line 90-13 (stably expressing Tet repressor) were used in this study. BSF cells were grown in HMI-11 medium and maintained in logarithmic phase [below 2 × 10^6^ cells/ml as described in Kalel et al. (2019)]. Genomically integrated stable transfections were performed with *Not*I-linearized plasmid constructs (pGN1-TbPEX11), which integrate into the spacer region of the ribosomal RNA repeat locus in the genome of cell line 90-13, and the clones were selected using blasticidin as described previously (Kalel et al., 2015). Expression of TbPEX11-GFP was confirmed with fluorescence microscopy following induction tests (data not shown) with a serial dilution of tetracycline, and minimal concentration (5 ng/ml) of tetracycline was used to achieve expression of the protein in more than 80% of the cells.

*T. brucei* BSF427 and compound dilutions were mixed in 1 : 1 (v/v) ratio to total volume of 200 μl, to reach final concentrations of 2 × 10^3^ cells/ml and 0.19–100 μM of inhibitors, in quadruplicates in 96-well plates. Culture medium with no cells was used as negative control and cultures without presence of compounds as positive control representing normal rate of cell growth. Cells were grown at 37°C in an incubator with humidified air containing 5% CO_2_ for 3 days. Cell viabilities were measured quantitatively using resazurin dye, by adding 25-µl resazurin (0.1 mg/ml in HBSS) to each well, and the mixture incubated for 6 h in the incubator. Fluorescence emission was detected at 570 and 585 nm after excitation at 530 nm, and fluorescence at 570 nm was subtracted from 585 nm. The inhibition curves were fitted with normalized fluorescence signal (in percentage) against concentration of compounds in Log10 scale using GraphPad Prism, and best fit was used for EC50 estimation. Chemical structures were drawn with ChemDraw 20.0.

### Immunofluorescence Microscopy

BSF 90-13 cells with genomically integrated PEX11-GFP were induced with 5 ng/ml of tetracycline and cultured overnight to initiate stable expression of PEX11-GFP. BSF427- and PEX11-GFP–expressing cells were treated for 24 h with 100 μM, 50 and 25 μM of each of the inhibitor, and DMSO was used as control. Compound-treated cultures with growth rates of about 50% compared to the DMSO control were harvested and stained for immunofluorescence and statistical analysis. Cells were fixed with 4% paraformaldehyde in PBS containing 250 mM sucrose for 20 min. Fixed cells were immobilized on adhesive slide (StarFrost^®^) pre-coated with 10% (v/v) of poly-l-lysine (Sigma-Aldrich^®^) in water after 1-h incubation at RT. Cells were permeabilized with 0.1% (v/v) Triton X-100 in PBS (pH7.4) for 15 min and blocked in buffer D [PBS supplemented with 1% (w/v) BSA and 0.25% (v/v) Tween 20] for 1 h. Anti-TbAldolase primary antibody was used at 1:500 dilution in buffer D for 1.5 h incubation. After five washes in PBS for 30 min, samples were treated with Goat anti-rabbit secondary antibody (1:200, v/v, Alexa Fluor™ 594). Samples were washed, dried, and mounted with Mowiol containing 4′,6-diamidino-2-phenylindole (DAPI). Immuno-stained cells were visualized with Zeiss Elyra microscopy. Pictures of stack 3 (of 5), rotation 1 (of 3), and phase 5 (of 5) were chosen for all control and compound-treated samples.

### Digitonin Fractionation

BSF427 cells were treated with compound **2**, compound **3**, or equivalent volume of DMSO for 24 h, and 2.4 × 10^6^ cells (16.5 μg protein) were harvested for each condition by centrifugation and washed once with homogenization buffer, containing 25 mM Tris-HCl (pH 7.4), 1 mM EDTA, 0.3 M sucrose, 1 mM DTT, and leupeptin (2 μg/ml). Pellets were resuspended in 420 μl of homogenization buffer and distributed evenly into four tubes each with 100 μl (4 μg protein). Digitonin (5% w/v stock in water) was diluted to corresponding concentrations, and 25 μl of which was added to the cell suspension to reach final concentration of 0.025×, 0.05×, 0.1×, or 2× (digitonin/protein, μg/μg). The mixture was incubated for 2 min at 37°C, vortexed for 10 s, and centrifuged (16,000 g) at 4°C for 15 min. 100 μl from the supernatant (solubilized fractions) was taken for Western blot analysis. Remaining pellet fractions were washed by adding homogenization buffer up to 125 μl and centrifuged again, supernatants were discarded, and pellets were resuspended in 100 μl for Western blotting.

### NMR Hit Validation Using Saturation Transfer Difference Experiments

NMR saturation transfer difference (STD) experiments (Mayer and Meyer, 1999) were carried out on a Bruker AVIII 600-MHz spectrometer equipped with a cryoprobe and a SampleJet auto sampler. One-dimensional (1D) and STD spectra were acquired at 298 K. Compounds were dissolved in DMSO-d6 to a final concentration of 50 mM. STD experiments with GST-TbPEX3, TbPEX19-His, and GST-His were performed in PBS (pH 7.4), 10% D_2_O at a protein concentration of 10 μM, and a ligand concentration of 300 µM. Saturation time and interscan delay within STD experiments were set to 2 and 2.5 s, respectively.

## Results

### Establishment of an AlphaScreen Assay for High-Throughput Screening of PEX3–PEX19 Interaction Inhibitors

**FIGURE 1:**
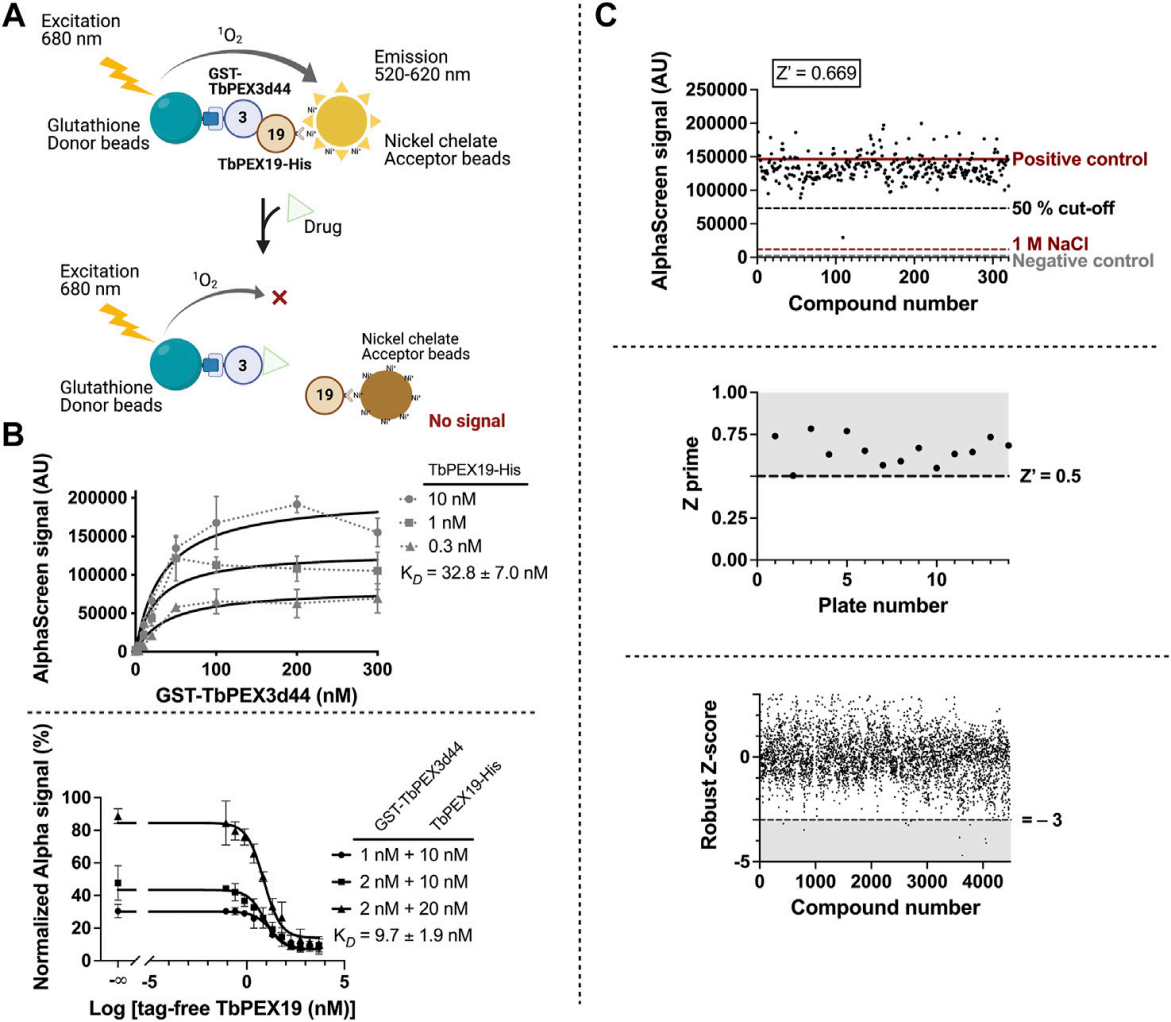
High-throughput TbPEX3–PEX19 inhibitor screening using AlphaScreen. **(A)** The screening assay with GST-TbPEX3d44 and TbPEX19FL-His. Interaction of the two proteins leads to different levels of light emission in the absence and presence of inhibitors, which can be quantitatively measured. **(B)** Binding of GST-TbPEX3d44 and TbPEX19FL-His was analyzed in a saturation assay **(upper panel)**; curves were fitted using one-site specific binding model with GraphPad. TbPEX19 was tested at three concentrations in independent saturation assays with serial dilution of TbPEX3, each of the data points represent the average of triplicates, and standard deviation is shown as vertical bars. An apparent *K*_*D*_ of 32.83 ± 7.04 nM was obtained from the three assays. **Lower panel** shows the binding affinity between GST-TbPEX19 and TbPEX19-His analyzed by competitive binding assay using tag-free TbPEX19. Curves were fitted using one-site homologous model which assumes tag-free TbPEX19 binds in the identical way as of TbPEX19-His to GST-TbPEX3d44. An apparent *K*_*D*_ of 9.7 ± 1.9 nM was estimated. **(C)** Compound screening assays were performed in 384-well plate format and individual plates contained following controls: negative control in the absence of protein complex showing background signal; positive control representing normal protein interaction, 1 M NaCl was used to completely dissociate the complex **(upper panel)**. Raw readings from an example plate with original AlphaScreen signal are shown in the upper panel using a 50% signal cutoff. Z prime value of each plate was calculated to ensure the assay reliability, with 0.5 ≤ Z’ ≤ 1 **(middle panel)**. Hits were selected using robust Z score; compounds giving robust Z score ≤ −3 were prioritized **(lower panel)**.

PEX19, the cytosolic receptor for PMPs, recognizes its cargo proteins through its C-terminal PMP binding domain. The N-terminal region of PEX19 mediates docking of the receptor cargo complex to the peroxisomal membrane via binding to PEX3. Thus, the PEX3–PEX19 interaction is the key step for the peroxisomal targeting and insertion of PMPs. Blocking this interaction will disrupt membrane biogenesis and, subsequently, matrix protein import, thus exerting lethal effect on trypanosomes. We previously showed that the recombinantly expressed GST-TbPEX3 lacking N-terminal 44 amino acids, which form the single-pass transmembrane domain (referred to as GST-TbPEX3d44 from here onward), interacts with the N-terminal 50 amino acid fragment of His-tagged TbPEX19 (TbPEX19_1-50aa_-His) in a pull-down assay (Kalel et al., 2019). To establish the high-throughput screening procedure for PEX3–PEX19 inhibitors, we utilized the AlphaScreen (PerkinElmer, Yasgar et al., 2016) technology, which we have previously used to identify PEX14–PEX5 inhibitors (Dawidowski et al., 2017). The AlphaScreen assay was established with purified GST-TbPEX3d44 and His-tagged full-length TbPEX19 (TbPEX19FL-His) (Figure 1A, Supplementary Figure S1A). Co-expressed and co-purified GST-TbPEX3d44 and TbPEX19-His was used for the compound screening assays. The complex co-migrated in size exclusion column with equimolar amounts of the components (Supplementary Figure S1B,C). These results indicate that the complexes are stable and that the tags do not interfere with the interaction. A saturation assay and two competitive assays were performed to confirm the stability and interaction between the purified proteins. The analysis of the interaction revealed apparent dissociation constants of *K*_*D*_ = 32.83 ± 7.04 nM (saturation assay) and *K*_*D*_ = 9.7 ± 1.9 nM (competitive assay using tag-free TbPEX19) (Figure 1B). An additional competitive assay was performed with TbPEX19-Strep, with apparent *K*_*D*_ of 3.7 ± 0.2 nM (Supplementary Figure S2). For the compound screening, the co-expressed and co-isolated complex of GST-TbPEX3d44 and TbPEX19FL-His was applied.

The assay was established in 384-well format with in-plate controls, including negative controls (no protein complex present) and positive controls (protein complex in the absence of chemical compounds). The two controls are indicative for the background noise and the signal without compound interference. As an additional negative control, dissociation of the PEX3–PEX19 interaction was achieved by incubation with 1 M NaCl (Ihrig and Obermann, 2017). We screened more than 4,000 compounds from the ChemBridge diversity library at a fixed concentration of 10 µM to test their capacity for inhibiting the TbPEX3–PEX19 interaction. An example of an individual assay, including the three controls and 320 compounds tested per 384-well plate is shown in Figure 1C (upper panel). The Z’ factor calculation considered positive (proteins present) and negative (no-proteins present) controls, as well as the corresponding dynamic range (Zhang et al., 1999). Therefore, it is regarded as a general approach for the evaluation and comparison for individual assays and an overview for all assays in our screenings, with a cutoff value of 0.5 (Figure 1C, middle panel). Robust Z-score was initially developed for RNAi screens. It is preferable for incorporating the variation among individual samples and, meanwhile, insensitive to the outliers (Chung et al., 2008; Birmingham et al., 2009). It utilizes median and median absolute deviation, and compounds with robust Z-score smaller than −3 were considered as causing significantly decreased signals from the majority (Figure 1C, lower panel).

**FIGURE 2:**
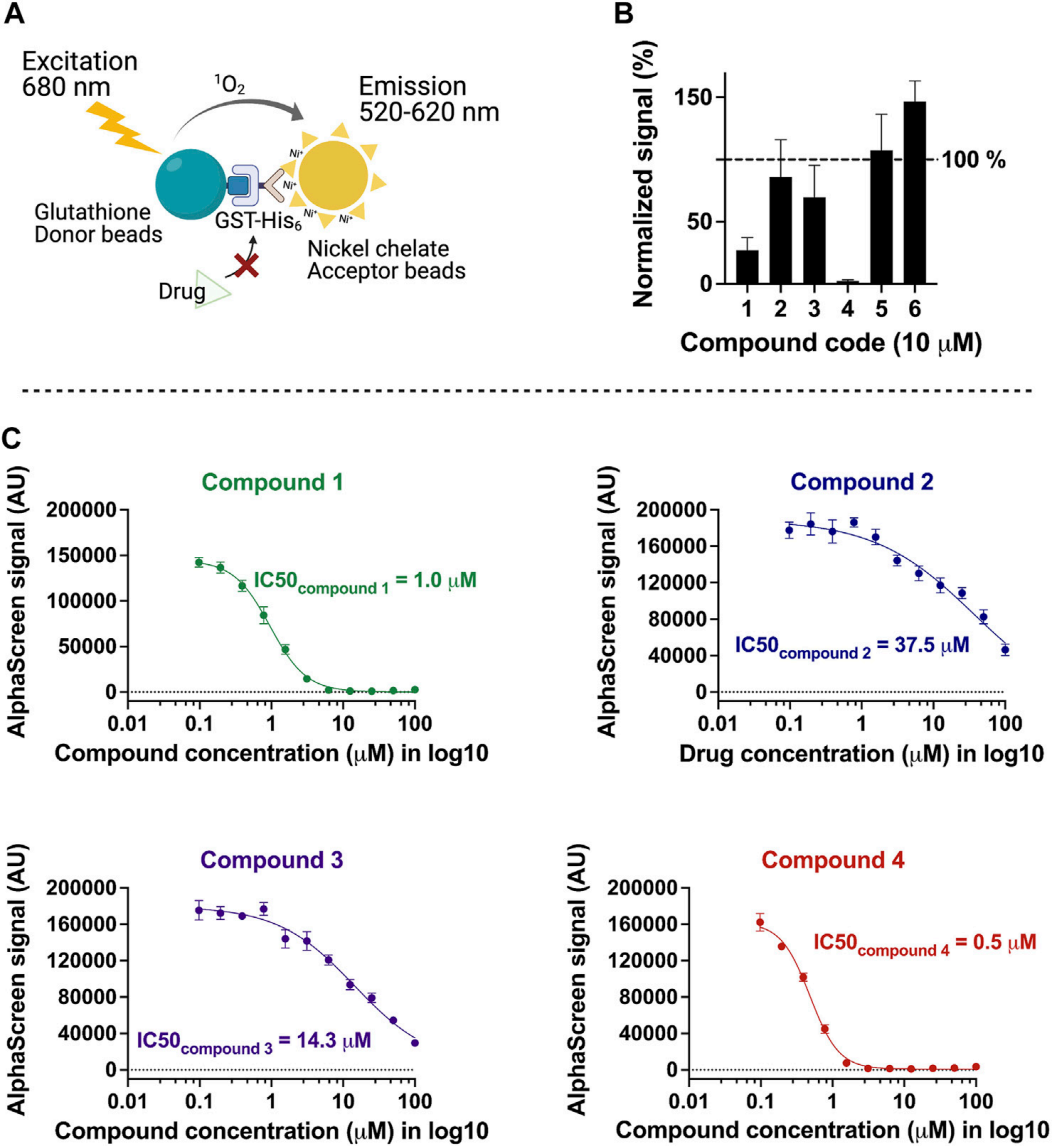
Characterization of inhibitors with the counter screen assay and dose-dependent response analysis. **(A)** Recombinant GST-His was used as a control to identify compounds that non-specifically reduce the AlphaScreen signal. **(B)** Normalized levels of compound-response using test (signal_TbPEX19-TbPEX3_) against control (signal_GST-His_), 10 μM of each compound was tested with TbPEX19–TbPEX3 complex or GST-His, four of six candidates (compounds **1**, **2**, **3**, and **4**) were selected for future analyses. **(C)** Dose-dependent response of the four compounds against TbPEX19–TbPEX3 interaction. IC50 values of the four compounds are 0.5, 1.0, 14.3, and 37.5 µM for compounds **4**, **1**, **3**, and **2**, respectively.

On the basis of the above criteria, six compounds were prioritized for further analysis. A counter-screen assay using GST-His was performed to elucidate whether these compounds interfere with the Alpha signal systematically, for example, by intrinsic fluorescence or unspecific binding to the affinity tags or the beads (Figure 2A). Ten micromolars of each compound in DMSO or DMSO alone were incubated with either TbPEX3–TbPEX19 complex or GST-His in parallel, and signal_compound_ was normalized to signal_DMSO_ in percentage for each assay condition. Signal_compound_ from GST-His was adjusted to 100% to allow comparison with the percent signal of TbPEX3–TbPEX19 (Figure 2B). Binding of two hits did not yield reproducible results, whereas the remaining compounds **1**, **2**, **3**, and **4** showed varied levels of specific inhibition of TbPEX3–TbPEX19 in comparison with GST-His. The four drug candidates showed a dose-dependent response in the TbPEX3–TbPEX19 interaction. The IC50 values (50% inhibitory concentration) of compounds **4**, **1**, **3**, and **2** were determined to be 0.5, 1.0, 14.3, and 37.5 µM, respectively (Figure 2C).

### Validation of the Hits Using an Independent *in vitro* Assay

**FIGURE 3:**
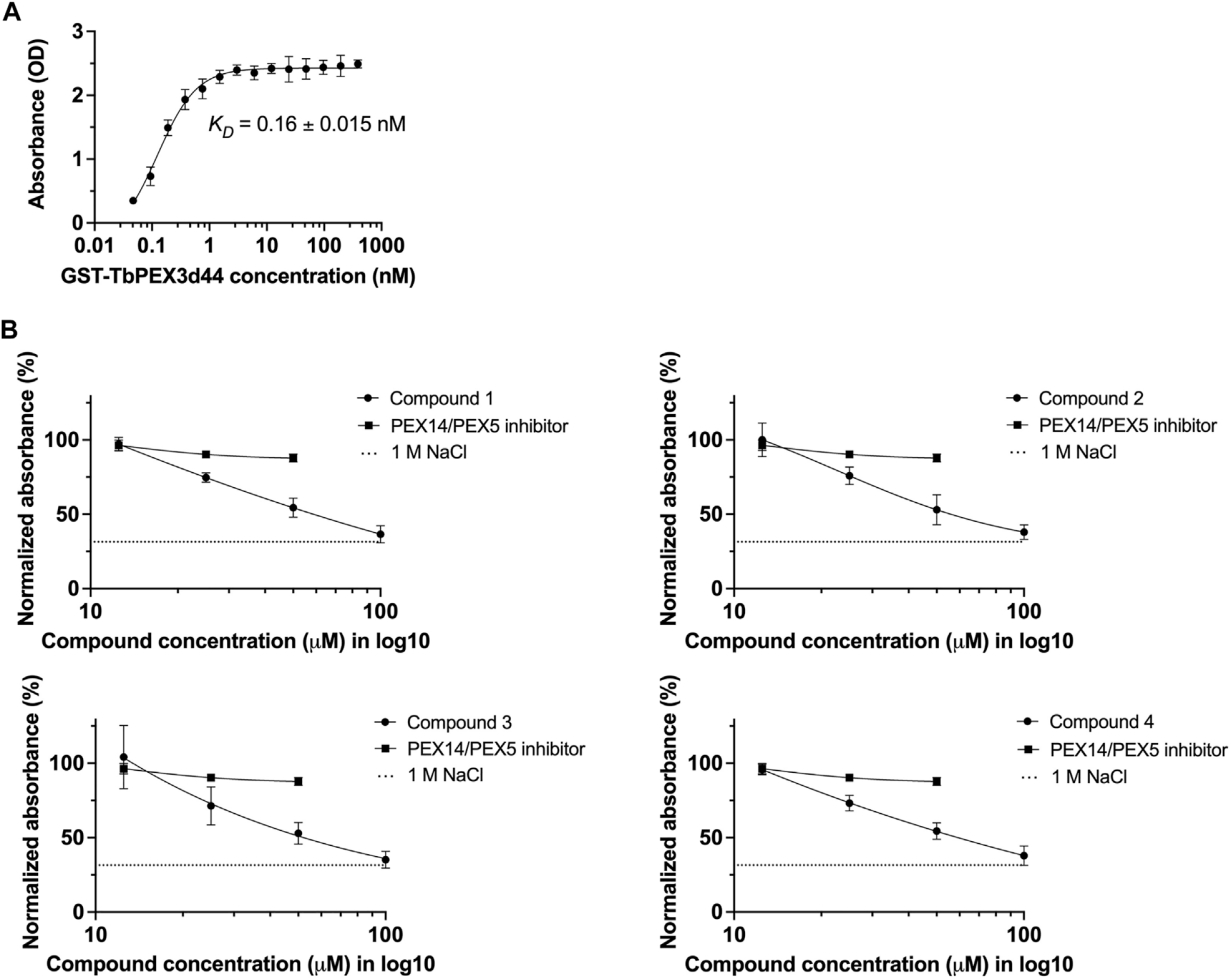
Validation of TbPEX3–TbPEX19 interaction inhibition using ELISA assays. **(A)** 1 µg of PEX19-His was coated to the wells of ELISA plate, blocked with BSA followed by incubation with different concentrations of GST-TbPEX3d44. Unbound PEX3 was removed, and bound PEX3 was detected using anti-GST primary antibody, horseradish peroxidase (HRP)–coupled secondary antibody, TMB as colorimetric substrate, and detection of absorbance at 450 nm. The binding curve of GST-TbPEX3d44 and TbPEX19FL-His was fitted using a simple 1 to 1 binding model and an apparent *K*_*D*_ value of 0.16 ± 0.015 nM was estimated (n = 2). **(B)** Dose-dependent inhibition of PEX3–PEX19 interaction by all four selected compounds was confirmed with ELISA assays. A TbPEX14–TbPEX5 inhibitor was used as a negative control. Incubation with 1 M NaCl abolished the interaction.

To confirm the inhibition of TbPEX3–TbPEX19 interaction by an independent assay, an ELISA assay was established. To determine the binding affinity, TbPEX19FL-His was coated to the wells of a microtiter plate, and different concentrations of GST-TbPEX3d44 were titrated. This saturation assay showed strong binding of TbPEX3 to TbPEX19, with an apparent *K*_*D*_ of 0.16 ±  0.015 nM (Figure 3A). The lower *K*_*D*_ value, when compared with AlphaScreen assay, is probably due to the dimerization of GST that causes amplification of signal via detection with anti-GST antibody. The presence of the dimerizing GST in the complex, detection via anti-GST antibody and secondary enzyme-coupled antibodies, which are used to detect ELISA signal, can lead to a considerable level of signal amplification independent of PEX19-binding and might be the cause for the lower *K*_*D*_ value. Therefore, the calculated binding constant can only be considered as apparent K_*D*_ value.

Next, we measured the dose-dependent response for the four identified compounds on the PEX3–PEX19 interaction using the ELISA. NaCl (1 M), which blocks protein–protein interactions (PPIs) (Ihrig and Obermann, 2017) and the TbPEX14–TbPEX5 inhibitor MAB-NH_2_ (Dawidowski et al., 2017) that should not block the PEX3–PEX19 interaction served as negative controls. Dose-dependent inhibition of the interaction was observed for all four compounds, whereas MAB-NH_2_ did not affect the interaction, thus demonstrating the specificity of the PEX3–PEX19 inhibitors (Figure 3B). About 50 μM of the compounds was required to achieve 50% of reduction in the normalized absorbance. Furthermore, these compounds were also tested on other protein complexes to investigate the possibility of unspecific inhibition. To this end, binding of TbPEX14-His and biotinylated TbPEX5 peptide was analyzed. None of the compounds affected the interaction up to 10 μM tested conditions, indicating that they do not block protein interactions in the ELISA assay unspecifically (Supplementary Figure S3).

### Hit Validation and Target Identification by NMR

**FIGURE 4:**
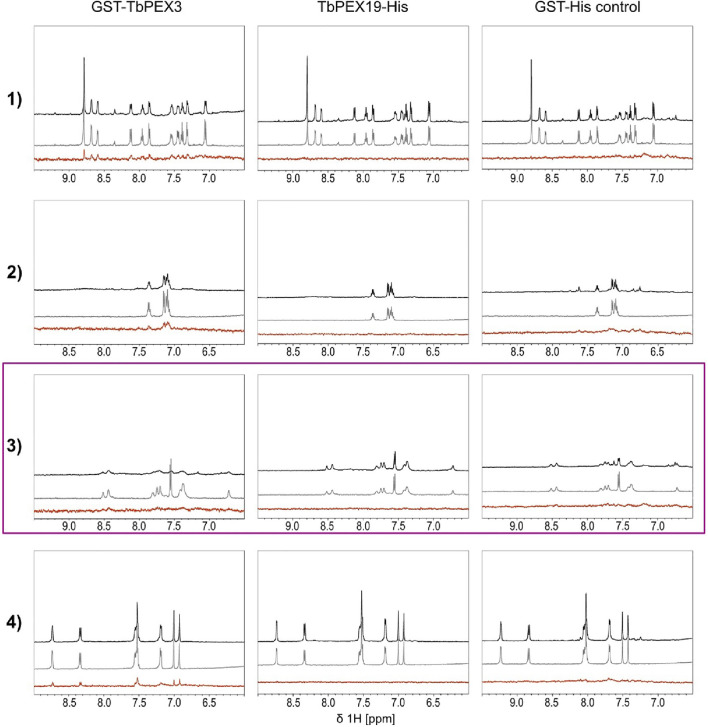
Saturation transfer difference (STD) NMR experiments**.** STD experiments were carried out with the compounds **1** to **4** and the targets GST-TbPEX3 **(left panels)**, TbPEX19-His **(middle panels)**, and as a GST-His control **(right panels)**. 1D spectra of compound in the presence and absence of protein are shown on black and gray, respectively. STD difference spectra of the compound in the presence of protein are shown in red. Compounds **1**, **2**, and **4** show strong STD signal with GST-TbPEX3 but not with TbPEX19 or GST representing good binding toward TbPEX3. Compound **3** shows strong line-broadening with GST-TbPEX3, and weak line-broadening when measured with the GST-His control.

We performed two independent NMR STD experiments to validate the hits (compounds **1**–**6**) and to identify which protein is directly targeted by the inhibitors. STD experiments were performed with GST-TbPEX3d44, TbPEX19-His, and GST-His alone. The level of confidence of binding is generally indicated by signal intensities in the STD difference spectra (Figure 4, red). In both analyses, compounds **1**, **2**, and **4** showed significant STD effects upon binding to TbPEX3 (Figure 4 and Supplementary Figure S5A, **GST-TbPEX3/left panels**) and not to GST-His, indicating that they directly bound to TbPEX3d44. In addition, compounds **5** and **6** also showed consistent STD effects for binding to TbPEX3 (Supplementary Figure S5B). In the initial NMR experiments, compound **3** showed notable STD signal with TbPEX19 and line-broadening with GST-TbPEX3d44 (Supplementary Figure S5A, row **3**). In the NMR analysis with optimized relaxation filter, compound **3** experienced line-broadening effect with GST-TbPEX3d44 and, to a much less extent, with GST-His (Figure 4, **TbPEX3/left panel**; **GST-His/right panel**). The higher level of line-broadening seen with compound **3** with GST-TbPEX3d44 may be explained by binding of compound **3** to both GST and TbPEX3d44.

### Anti-trypanosomal Activity and Cytotoxicity Analysis

**FIGURE 5:**
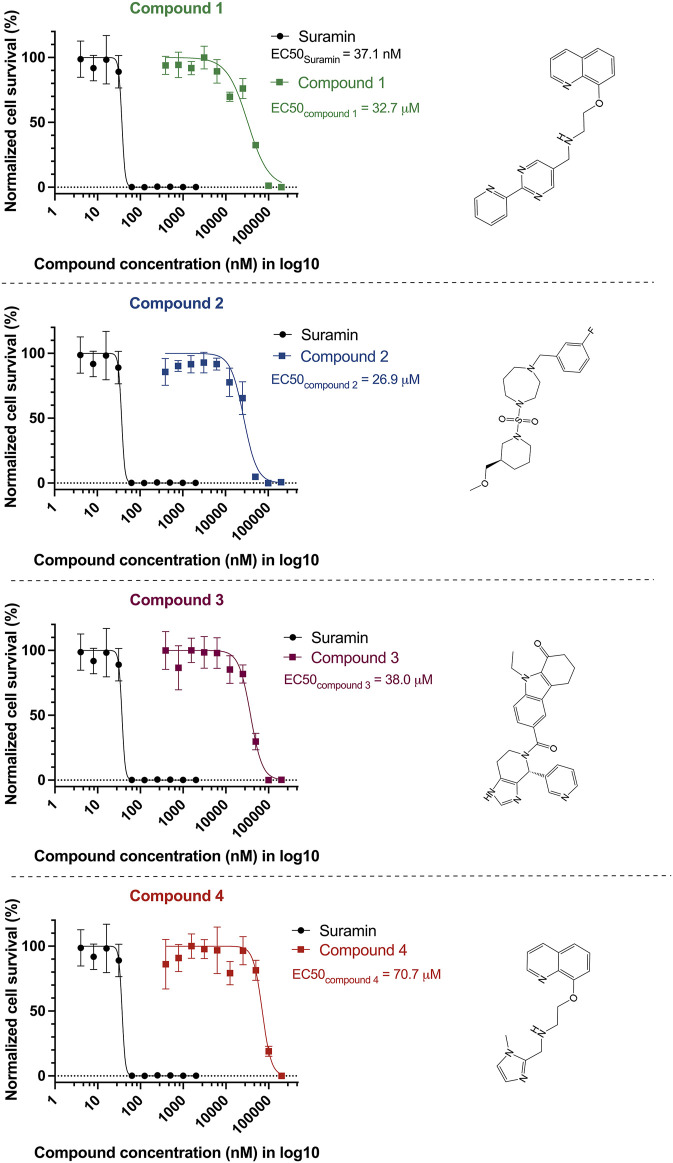
Anti-trypanosomal activity of the inhibitors. Bloodstream form of wild-type *T. brucei* parasites (BSF427) was treated with serial dilutions of PEX3–PEX19 inhibitors or suramin as a positive control. After 3 days of incubation, cell viability was estimated using resazurin-based assay. Cell survival levels for all compound-treated conditions were normalized and shown in percentage plotted against compound concentration in Log_10_ scale, EC50 of suramin is 37 nM (black curve). Survival percentage for other compounds is drawn in curves of corresponding colors; the EC50 values of the four compounds are as follows: 33 μM (compound **1**, green), 27 μM (compound **2**, navy blue), 38 μM (compound **3**, purple), and 71 μM (compound **4**, orange). The corresponding structure of the compounds is shown on the right.

We tested the activity of the PEX3–PEX19 inhibitors against cultured BSF *T. bruce*i parasites. Parasites were treated with increasing concentrations of the compounds, and cell viability was estimated using resazurin-based assay after 3 days of incubation (Figure 5). The potent inhibitor suramin was used as a positive control, resulting in a half-maximal effective concentration of 37 nM (concentration leading to 50% reduction in cell survival, EC50). The identified compounds **2** (navy blue) and **1** (green, which exhibited one of the lowest IC50 values in the AlphaScreen assays) showed EC50 values of 27 and 33 μM, respectively. Compound **3** (purple) showed an EC50 of 38 μM, and compound **4** (red) exhibited an EC50 of 71 μM.

**FIGURE 6:**
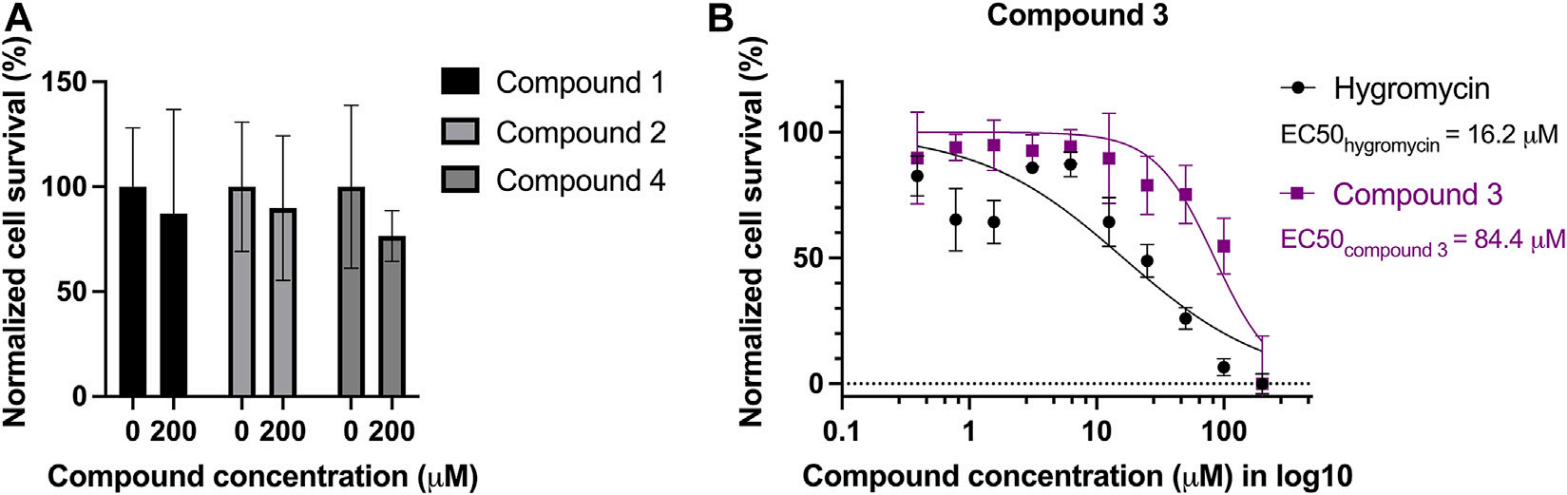
Cytotoxicity test of the four compounds on HepG2 cells. Mammalian cell line HepG2 was treated with serial dilutions of the four compounds: **(A)** compound **1**, **2**, and **4** led to minor reduction of the survival rate, ranging from 75% to 90% at 200 μM, in comparison to the survival rate without treatment. **(B)** Compound **3** is toxic to HepG2 with an EC50 of 84.4 µM. Hygromycin was used as a control for an active drug, and it has an EC50 of 16.2 µM.

The compounds were also tested against human cells using a similar assay to estimate cytotoxicity of the compounds. HepG2 cells were treated with the four selected compounds with serial dilutions of up to 200 µM. No dose-dependent response curve could be fitted with cells incubated with compound **1**, **2**, and **4** but treated cells showed 75%–90% of survival at 200 µM (Figure 6A), whereas compound **3** seems to be toxic to the cells at high concentrations, with an EC50 of 84.4 µM. Hygromycin served as an active drug control (Figure 6B).

### Trypanosomal On-Target Activity of the Compounds

**FIGURE 7:**
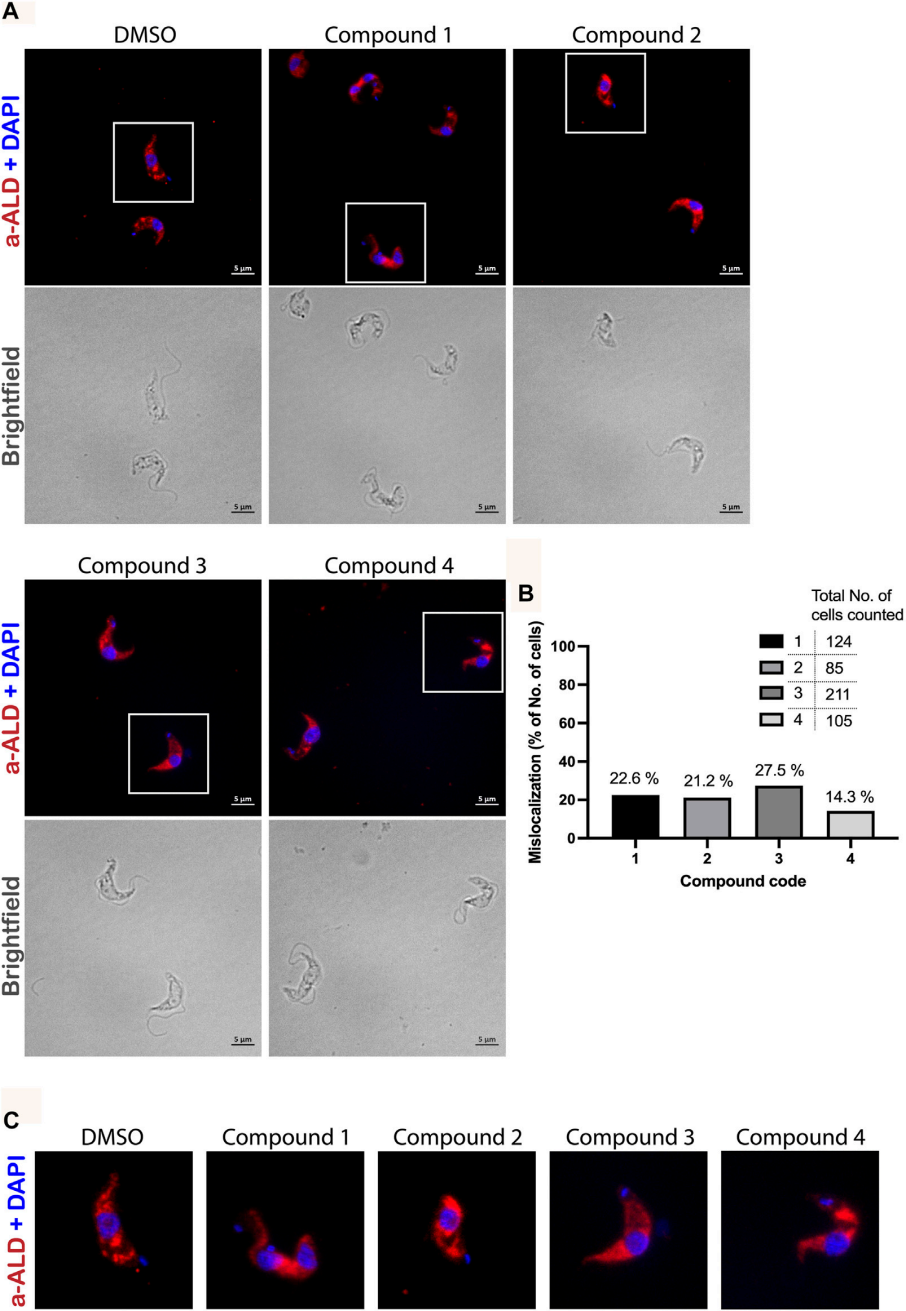
Immunofluorescence microscopy analysis of the effect of PEX3–PEX19 inhibitors on glycosomes. **(A)** Wild-type *T. brucei* bloodstream form parasites were treated with DMSO or inhibitors for 24 h. Compound-treated cultures with 50% of the growth rate compared to the DMSO control were fixed followed by staining with anti-TbAldolase primary antibody and Alexa Fluor 594–labeled secondary antibody. (a-ALD, red; DAPI-labeled nucleus and kinetoplast, blue). Corresponding bright-field images are shown in the lower panels. A punctate pattern indicated the localization of aldolase in glycosomes (DMSO). Different levels of mislocalization of aldolase to the cytosol were noticed in each of the compound-treated samples. **(B)** Numbers of cells showing aldolase mislocalization were counted. About 20%–30% of cells treated with compounds **1**, **2**, and **3** and 15% of compound **4** treated cells showed aldolase mislocalized to the cytosol. **(C)** Images of individual cells, which are marked by white boxes in **(A)**, were 2X magnified for better illustration.

We performed immunofluorescence microscopy analysis to evaluate the effects of PEX3–PEX19 inhibitors on glycosomes. Cells were treated with the compounds or with DMSO alone as control. Treated cells were fixed, permeabilized, and immunolabeled using antibodies against glycosomal marker enzyme aldolase. Fluorescence microscopy revealed different levels of mislocalization of the glycosomal matrix marker enzyme aldolase when comparing to the DMSO-treated cells (Figure 7A). Cells marked with white boxes are zoomed in for better illustration (Figure 7C). In DMSO-treated sample, aldolase labeling indicates a typical punctate pattern in vast majority of the cells. In contrast, the compound-treated cells exhibited a diffuse cytosolic staining of aldolase. In particular, compound 1 caused the well-recognized diffused pattern of aldolase labeling (Figure 7A). Compound **2** caused large numbers of cells that showed an aberrant morphology, and cells with normal shape exhibited mislocalization of aldolase to the cytosol. Compound **3**–treated cells mostly retained their cell morphology, and partial mislocalization of aldolase to the cytosol is observed. Compound **4** caused patches of stained aldolase, suggesting clustering of the glycosomes. The slight difference in the compound-treated phenotypes could be due to the low solubility and hydrophobicity of the compounds, which lead to a distinctive diffusion of the compounds after being taken up by the cells and, hence, a localized or varied level of exposure to the glycosomes. For statistical analysis, numbers of cells analyzed for each compound treated condition are shown in Figure 7B. Although the DMSO control showed no mislocalization, about 23% (compound 1), 21% (compound 2), 27% (compound 3), and 14% (compound 4) of cells treated with corresponding compounds were showing a diffuse labeling pattern of aldolase, indicative of mislocalization of glycosomal enzyme to the cytosol.

Moreover, when treating PEX11-GFP–expressing BSF cells with compound 2 (Supplementary Figure S4), it was observed in some cells that not only matrix proteins but also PEX11 were partly mislocalized to cytosol, whereas PEX11 was partly still glycosomal. When cells were treated with compound **3** (Supplementary Figure S4), mislocalization of both PEX11 and matrix proteins (aldolase and hexokinase) was even more pronounced with no obvious glycosomal localization of PEX11.

**FIGURE 8:**
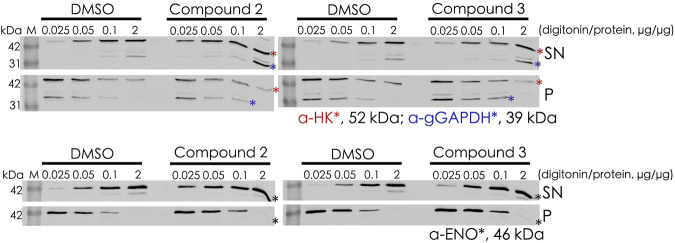
Digitonin fractionation of compound 2– and compound 3–treated BSF cells. Digitonin-solubilized fractions are indicated as supernatant (SN), and the non-solubilized fractions are indicated as pellet (P). Four concentrations of digitonin treatment were used to investigate the mislocalization of matrix proteins, and hexokinase (HK) and glyceraldehyde 3-phosphate dehydrogenase (gGAPDH) are labeled with red and blue asterisks, respectively **(upper panel)**. Enolase (ENO) as the cytosolic marker is indicated by a black asterik **(lower panel)**. At the lowest level of digitonin treatment (0.025 μg of digitonin/μg of total protein), HK and gGAPDH were released from compound-treated cells to a greater extent than from the corresponding DMSO control cells, indicating mislocalization of these proteins to the cytosol upon treatment.

Digitonin fractionation experiments (Figure 8) were performed as an independent method to investigate the mislocalization of the glycosomal matrix proteins. Compound **2–** and compound **3**–treated BSF cells were harvested and solubilized at different concentrations of digitonin varied from 0.025× to 2× (digitonin/protein, v/v). When compound-treated cells were incubated with the lowest concentration of digitonin (0.025 μg of digitonin/μg of total protein), higher level of hexokinase (HK) and glycosomal GAPDH were released into the supernatant fractions in comparison to the corresponding DMSO control. HK behaved similarly to the cytosolic marker enolase, indicating that the treatment with compounds **2** and **3** results in mislocalization of the matrix protein into cytosol.

## Discussion

Existing trypanocidal drugs have been extensively studied but novel compounds with potential in treating these infections are urgently required. Inhibition of glycosomal compartmentation affects several essential metabolic pathways and thus provides an attractive drug target. We previously developed small-molecule inhibitors of PEX14–PEX5 PPI that block glycosomal matrix protein import and kill *T. brucei* parasites (Dawidowski et al., 2017, 2020). These inhibitors also showed therapeutic efficacy upon oral delivery in animal models of infection (Dawidowski et al., 2017). Recently, we and others identified *Trypanosoma* PEX3, a long-sought docking factor for the membrane protein import receptor PEX19 (Banerjee et al., 2019; Kalel et al., 2019). This discovery enabled the exploration of a therapeutic approach targeting the PEX3–PEX19 interaction as a candidate for drug target to identify small molecules that disrupt glycosome biogenesis and kill parasites.

In this study, we report on a high-throughput, 384 well-plate compatible, AlphaScreen-based screening method to identify inhibitors of the TbPEX3–PEX19 interaction. For discovering PPI modulators, use of chemically diverse compound libraries are preferable to maximize the chances of matching the PPI target (Lu et al., 2020). We screened 4,480 compounds (Supplementary File S1) from the ChemBridge Diversity library, which led to the identification of six initial hits after applying statistical quality control and hit selection criteria. Interference of the compounds with assay reagents or readout can lead to false positives, and these can be distinguished using counter-screening (Schorpp et al., 2014). Our counter-screen using GST-His identified four compounds that displayed higher specificity targeting the TbPEX3–TbPEX19 interaction. Independent ELISA-based biochemical assays further validated that the shortlisted compounds specifically blocked TbPEX3–PEX19 interaction *in vitro*.

Cellular assays showed that all four compounds exhibit trypanocidal activity against BSF *Trypanosoma* parasites. Compound **2** showed the highest trypanocidal activity with EC50 of 27 μM (concentration leading to 50% cell death). The compounds identified in this study represent chemical starting points like the first TbPEX14–PEX5 inhibitor compound **1** with EC50 of 21 μM (Dawidowski et al., 2017). As the specific target and structure were known, this PEX14–PEX5 inhibitor was successfully optimized to potent trypanocidal compounds with nanomolar EC50, using a structure-guided approach. Similar optimization of the PEX3–PEX19 inhibitors could enhance their trypanocidal efficacy in future.

The compounds showed no apparent toxicity to human cells, except compound **3**, which showed cytotoxicity with EC50 of 84 μM. However, patient-derived PEX3 defective human fibroblast cells (Ghaedi et al., 2000; Muntau et al., 2000) are viable in cell culture. This suggests that cytotoxicity of compound **3** could be non-specific and not PEX3–PEX19 related.

We also performed hit validation and target confirmation using NMR. The STD effects observed for compounds **1**, **2**, and **4** with TbPEX3 or TbPEX19 are in good agreement with the trypanocidal activity and the performance of the compounds in inhibiting the PEX19-PEX3 interaction. It is less conclusive to which proteins compound **3** binds; based on the second NMR analysis with optimized settings, it is possible that the compound binds to TbPEX3 and to a less extent to GST. Although compounds **5** and **6** did not inhibit TbPEX3–PEX19 interaction, they show STD effect with TbPEX3. It is possible that compounds **5** and **6** bind to TbPEX3 distant from the binding interface with TbPEX19 and that this has no significant effect on the interaction between these proteins. Activities of the compounds in various assays in this study are summarized in Table 2.

**TABLE 2:** Comparison of drug properties from *in vitro* and *in vivo* analyses.

Compound	TbPEX3-19 affinity (IC50, μM)	*T. brucei* toxicity (EC50, μM)	Target protein (NMR analysis)	Human cell cytotoxicity (EC50, μM)
1	1.0	33	TbPEX3	>> 200
2	37.5	27	TbPEX3	>> 200
3	14.3	38	TbPEX19	84
4	0.5	71	TbPEX3	>> 200

Finally, immunofluorescence analysis and digitonin fractionation showed that these compounds disrupt glycosome biogenesis, leading to mislocalization of glycosomal enzyme and parasite death. Inhibition of the PEX3–PEX19 interaction would disrupt import of PMPs, including those involved in matrix protein import. Even partial mislocalization of glycosomal enzymes is toxic for trypanosomes, and thus, parasites would be killed before mislocalization of PMPs is evident. Clustering of glycosomes was also seen in some cells, which could be due to imbalance of membrane protein targeting. Clustering of glycosomes was also seen in trypanosomes overexpressing GFP-tagged TbPEX16, but it is also frequently seen in normal cells (Kalel et al., 2015; Hughes et al., 2017). Considering the cytotoxicity of compound **3** and the NMR analysis suggesting a potential GST binding, this compound could be less specific in comparison to compound **2**. To this extent, compound **2** is in higher priority for future structural-based optimization. Structural studies have shown that PEX3 provides the binding surface/pocket for the binding of the N-terminal helix in PEX19 (Sato et al., 2010; Schmidt et al., 2010). Therefore, it is more likely that the inhibitors of the PEX3–PEX19 interaction identified in this study bind to PEX3, block the binding pocket, and thereby prevent docking of PEX19.

The physicochemical properties of the four compounds are in consistence with Lipinski’s rule of 5 (Supplementary Figure S6). These parameters describe the permeability and solubility of the compounds and suggest that these compounds exhibit promising drug-like properties. The quinoline and triazolopyrimidine scaffolds have been reported as drug-like in the *in vitro* assays and a wide range of *in vivo* anti-microbial activities. To be specific, three chloroquinoline derivatives, which were previously evaluated as anti-malarial compounds have been identified as sub-micromolar inhibitors of intracellular *T. cruzi* (Magdaleno et al., 2009; Fonseca-Berzal et al., 2014). It has also been reported that triazolopyrimidine derivatives lead to nanomolar range of EC50 in *T. brucei* and *T. cruzi*, and three triazolopyrimidines are showing better suppression of the disease in *T. cruzi* mouse infection model than the front-line drug benznidazole (Nagendar et al., 2019).

Our study demonstrates that PEX3–PEX19 interaction is a druggable target in *Trypanosoma* and provides a high-throughput compatible screening platform for further screening of the inhibitors of this PPI. Structural investigations such as co-crystallization of the protein-compound complex would certainly aid in the future structural-guided optimization of these compounds to develop new therapies against trypanosomiasis and leishmaniasis.

## Data Availability

The original contributions presented in the study are included in the article/Supplementary Material, further inquiries can be directed to the corresponding authors.
