# Supplementary material for: Novel Trypanocidal Inhibitors that Block Glycosome Biogenesis by Targeting PEX3–PEX19 Interaction
Source: Front Cell Dev Biol. 2021 Dec 20;9:737159. doi: 10.3389/fcell.2021.737159 (PMC8721105; doi:10.3389/fcell.2021.737159)
Supplement: Supplementary file 1 [file Image5.PDF]

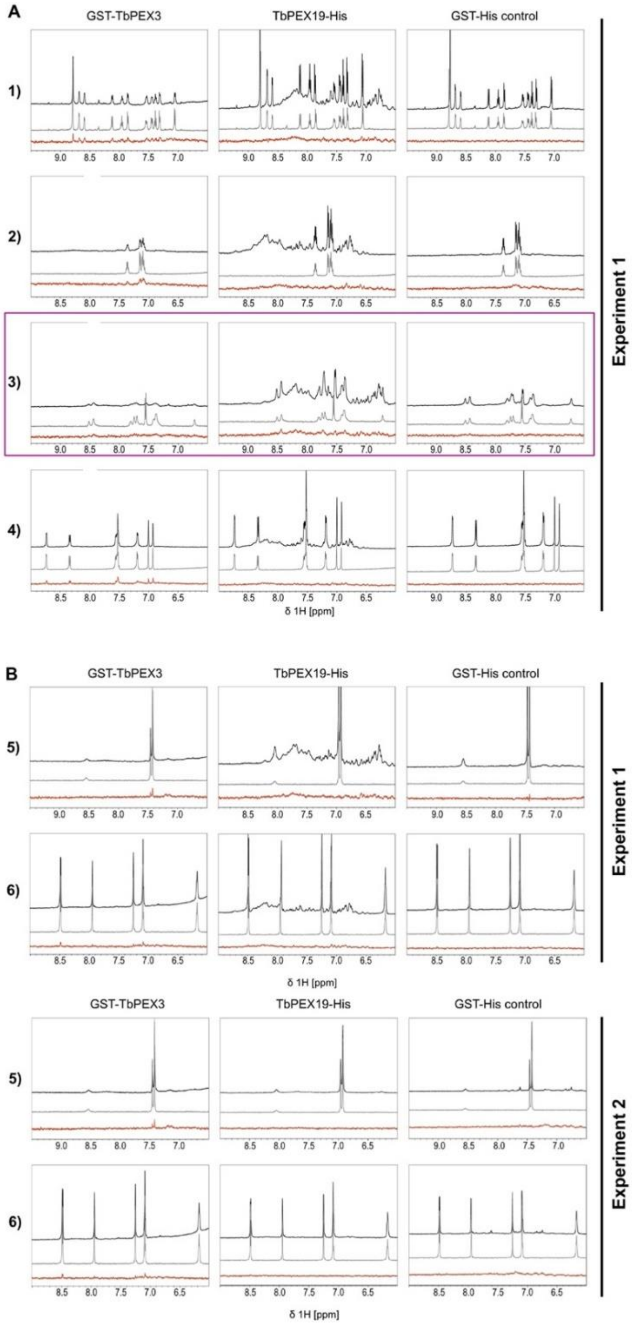

Supplementary figure 5. Saturation transfer difference (STD) analyses from NMR experiments 1 and 2. (A) Experiment 1. Compound **1**, **2**, **3**, and **4** (as labelled in the left, from top to the bottom in numeric order) were used to treat GST-TbPEX3d44 (left panels), TbPEX19-His (middle panels) and GST-His (right panels). 1D spectra of compound in the presence and absence of protein is shown in black and grey, respectively. STD difference spectra of the compound in the presence of protein are shown in red. Confidence of binding is generally indicated by the peak intensity (signal to noise). Compound **1**, **2** and **4** showed strong STD signal with GST-TbPEX3d44 but neither TbPEX19-His nor GST control. Compound **3** show STD signal towards TbPEX19 and experience strong line-broadening with measured with GST-TbPEX3. **(B)** Compounds **5** and **6** were tested in both experiment 1 (upper panels) and 2 (lower panels). The two compounds show STD signal with GST-TbPEX3 in both experiments.
