# Supplementary material for: Novel Trypanocidal Inhibitors that Block Glycosome Biogenesis by Targeting PEX3–PEX19 Interaction
Source: Front Cell Dev Biol. 2021 Dec 20;9:737159. doi: 10.3389/fcell.2021.737159 (PMC8721105; doi:10.3389/fcell.2021.737159)
Supplement: Supplementary file 2 [file Image6.PDF]

Compound	Structure	clogP	tPSA	RB	Hacc	Hdon	MW
**1**	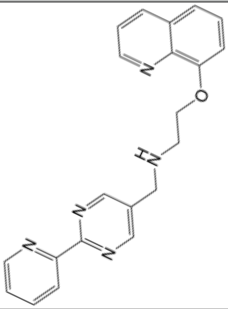	2.22	72.82	7	6	1	357.4
**2**	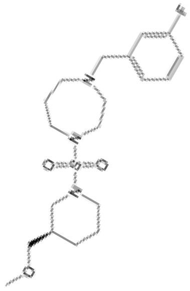	0.57	53.09	4	4	0	399.5
**3**	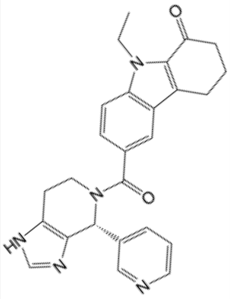	0.78	83.88	3	4	1	439.5
**4**	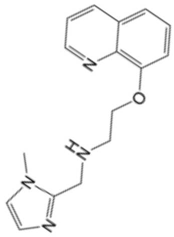	1.18	51.97	6	4	1	282,3
**Lipinski values**	-	≤ 5	≤ 140	≤ 10	≤ 10	≤ 5	≤ 500

Supplementary figure 6. Structure and physicochemical properties of the prioritized 4 compounds. Physicochemical properties of the compounds provided by the compound library supplier (ChemBridge) are listed. Partition coefficient (clogP), topological polar surface area (tPSA, Å^2), rotatable bonds (RB), hydrogen bonding (Hacc, hydrogen bond acceptor; Hdon, hydrogen bond donor) and molecular weight (MW, g/mol). Lipinski values as reference are shown in the bottom row.
