# Supplementary material for: Novel Trypanocidal Inhibitors that Block Glycosome Biogenesis by Targeting PEX3–PEX19 Interaction
Source: Front Cell Dev Biol. 2021 Dec 20;9:737159. doi: 10.3389/fcell.2021.737159 (PMC8721105; doi:10.3389/fcell.2021.737159)
Supplement: Supplementary file 3 [file Image4.PDF]

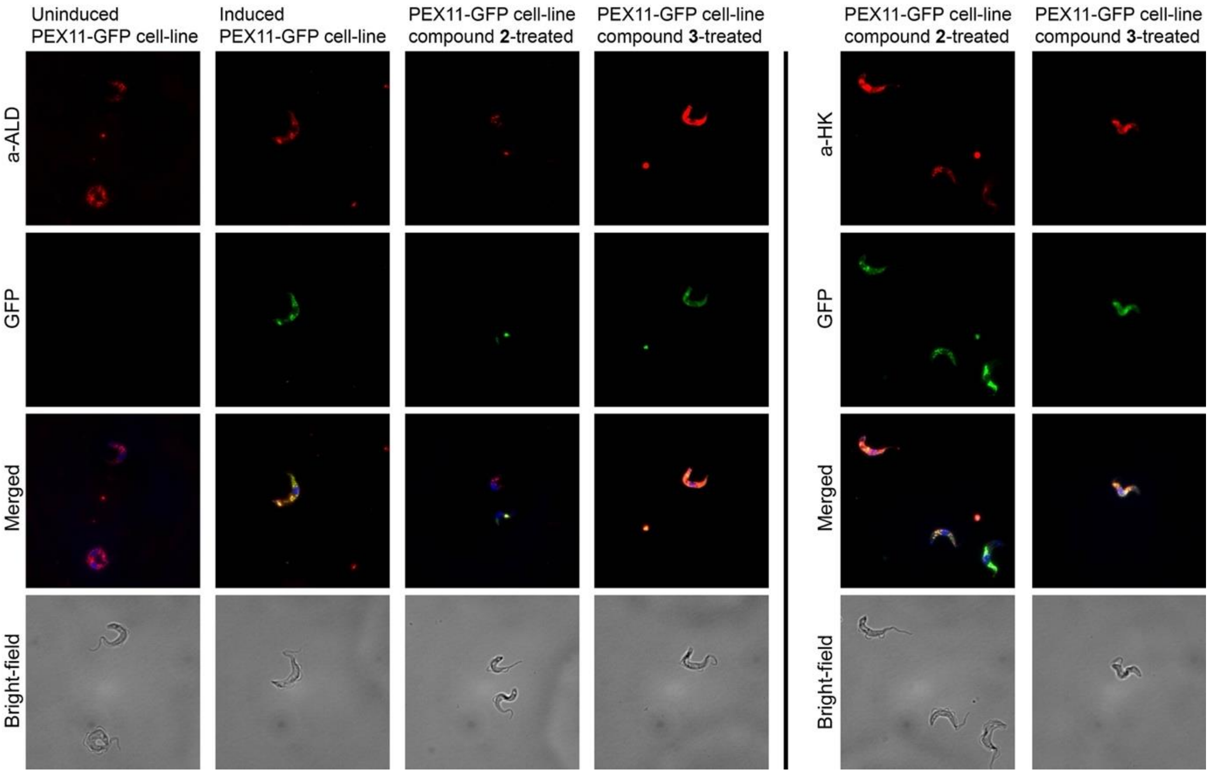

Supplementary figure 4. Glycosomal membrane and matrix protein localization in BSF cells expressing TbPEX11-GFP. BSF cells with integrated *TbPEX11-GFP* gene under the control of tetracycline operator were selected and maintained with blasticidin (row 1 from the left, vertical). Expression of PEX11-GFP in over 80 % of the cells was achieved by using 5 ng/ml of tetracycline (row 2). Analyzed marker proteins are indicated on the left: matrix marker (ALD/HK, red), GFP (green), merged (matrix marker; GFP; DAPI, blue) and brightfield. PEX11-GFP expressing cells were treated with compound **2** (row 3 and 5) and compound **3** (row 4 and 6). Cells were stained with anti-aldolase (a-ALD) and anti-hexokinase (a-HK) to monitor matrix protein localization. GFP fluorescence was used as the indicator of PEX11-membrane protein localization. Compound **2** caused aggregation of glycosomes indicated by PEX11-GFP and aldolase localization (row 3). In row 5, compound **2**-treated cells show a partial mislocalization of PEX11 to the cytosol (top left cell), with remaining punctate pattern of glycosome. Compound **3** caused mislocalization of both PEX11-GFP and matrix proteins (row 4, ALD; row 6, HK) to the cytosol with no obvious glycosomal localization of PEX11.
