# Supplementary material for: Novel Trypanocidal Inhibitors that Block Glycosome Biogenesis by Targeting PEX3–PEX19 Interaction
Source: Front Cell Dev Biol. 2021 Dec 20;9:737159. doi: 10.3389/fcell.2021.737159 (PMC8721105; doi:10.3389/fcell.2021.737159)
Supplement: Supplementary file 4 [file Image2.PDF]

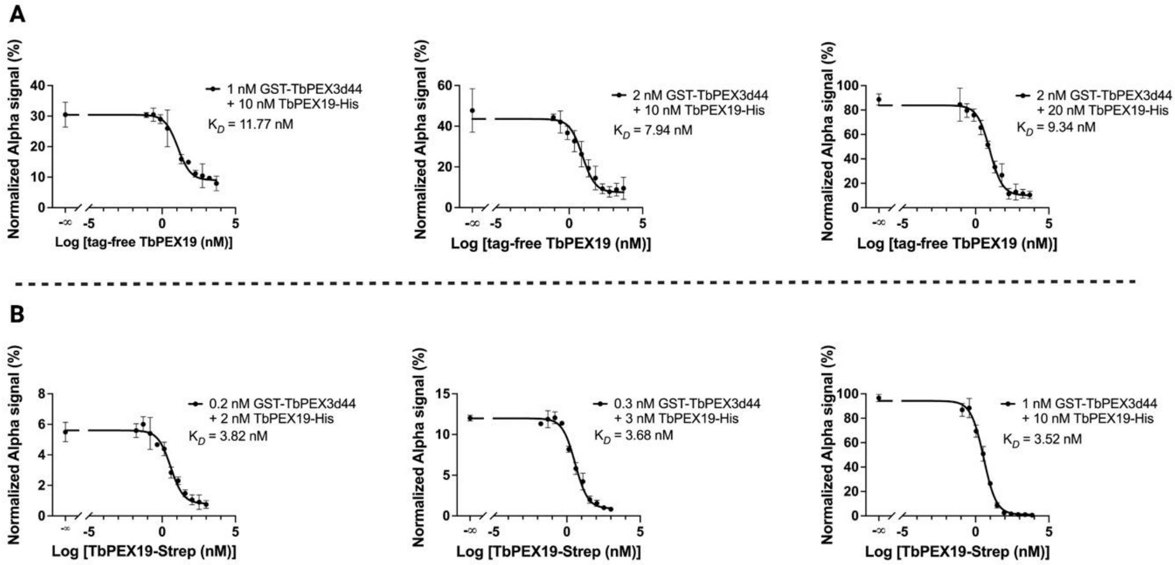

Supplementary figure 2. Competitive binding assays using tag-free TbPEX19 and TbPEX19-Strep. (A) GST-TbPEX3 and TbPEX19-His was used in three independent assays in molar ratios of either 1:10 or 1:5 (each with three technical replicates) as indicated. Raw Alpha signal output was normalized for comparison. Tag-free TbPEX19 was titrated (0 – 5 μM) and added to the interaction to compete with TbPEX19-His for binding. The competitive curve was fitted using GraphPad and a K_D of 9.7 $\pm$ 1.9 nM was estimated. **(B)** GST-TbPEX3d44 and TbPEX19-His was used for the assays in a molar ratio of 1:10 as indicated. TbPEX19-Strep (0 – 7.2 μM) was added to bind GST-TbPEX3d44 and to compete for binding of TbPEX19-His. The K_D was estimated to be 3.7 $\pm$ 0.2 nM.
