# Supplementary material for: Novel Trypanocidal Inhibitors that Block Glycosome Biogenesis by Targeting PEX3–PEX19 Interaction
Source: Front Cell Dev Biol. 2021 Dec 20;9:737159. doi: 10.3389/fcell.2021.737159 (PMC8721105; doi:10.3389/fcell.2021.737159)
Supplement: Supplementary file 5 [file Image3.PDF]

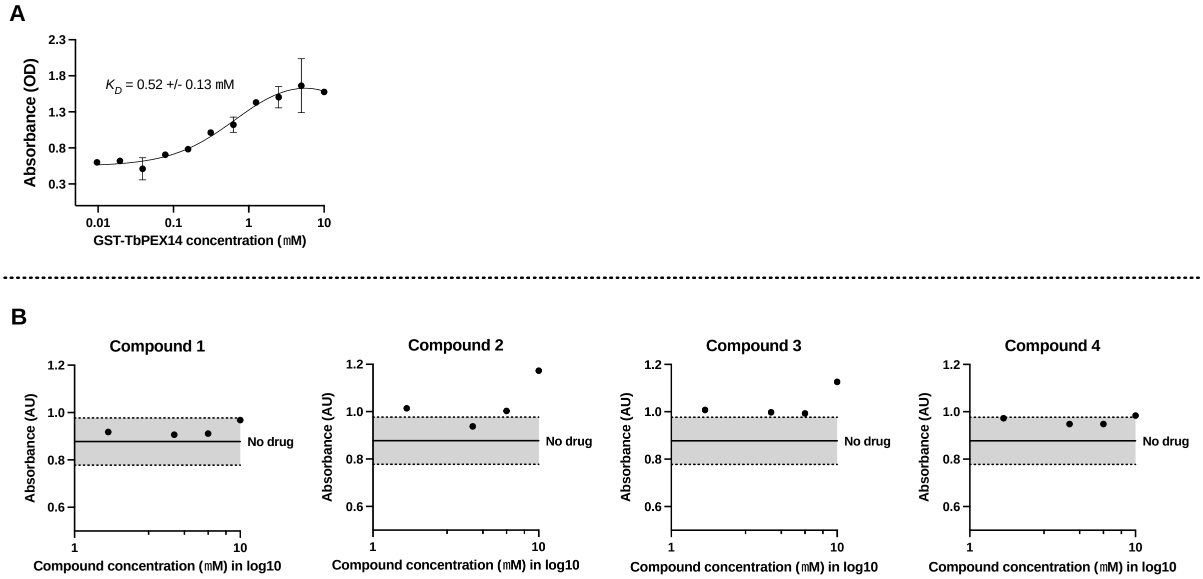

Supplementary figure 3. Testing of compounds on inhibiting formation of the TbPEX14-TbPEX5 complex. (A) The analysis of the interaction of GST-TbPEX14 (residue1-84) and Biotin-TbPEX5^{pep} revealed a K_D of 0.52 μM. **(B)** No dose-dependent response is observed for the four drugs at concentrations up to 10 μM.
