# Supplementary material for: Novel Trypanocidal Inhibitors that Block Glycosome Biogenesis by Targeting PEX3–PEX19 Interaction
Source: Front Cell Dev Biol. 2021 Dec 20;9:737159. doi: 10.3389/fcell.2021.737159 (PMC8721105; doi:10.3389/fcell.2021.737159)
Supplement: Supplementary file 7 [file Image1.PDF]

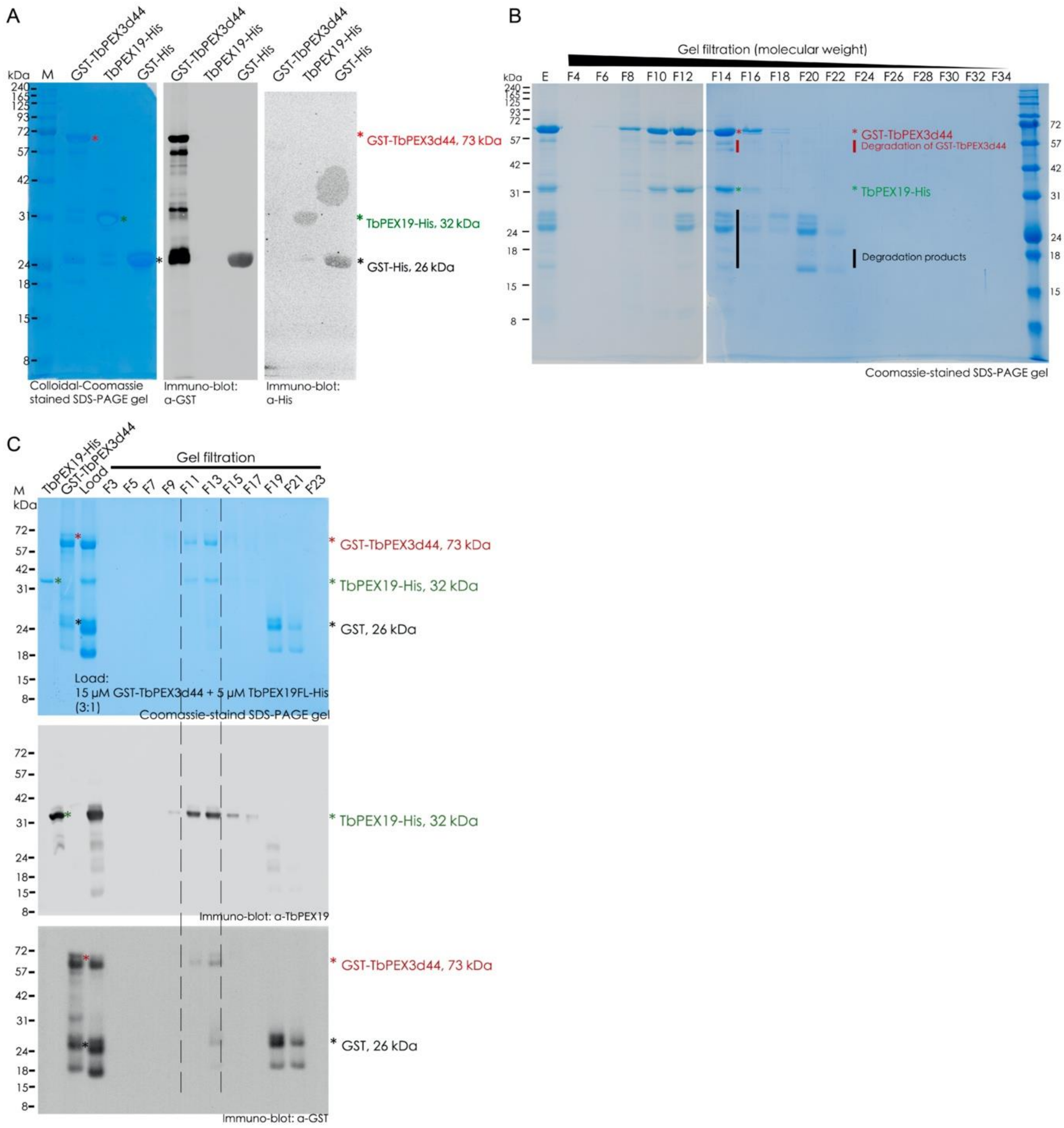

Supplementary figure 1. Protein preparation for compound screening and NMR analysis. (A) Proteins used for NMR studies were separately purified. Identities of the proteins were confirmed by immunobloting using anti-GST and anti-His antibodies (GST-TbPEX3d44, red; TbPEX19-His, green; GST-His, black). **(B)** GST-TbPEX3d44 and TbPEX19-His are co-expressed and purified by glutathione agarose beads. The eluate that contained the co-purified complex (E, elution) was further fractionated by size exclusion chromatography to remove impurities. Alternate fractions from F4 to F34 were analysed by SDS-PAGE. GST-TbPEX3d44 (red asterisk, 73 kDa) and TbPEX19-His (green asterisk, 32 kDa) co-migrated in F10 – F14 with equimolar amounts. A GST contamination is indicated by the black asterisk (26 kDa). **(C)** Complex formation of the individually purified proteins was analysed by size exclusion chromatography. GST-TbPEX3d44 and TbPEX19-His were pre-incubated in a 3:1 molar ratio, the mixture (Load) was fractionated by gel filtration. Eluted fractions were analyzed by SDS-PAGE (colloidal-coomassie stained gel, upper panel). The complex of GST-TbPEX3d44 and TbPEX19-His was co-migrated in F11 – F13. Protein identities were confirmed by immunoblotting with rabbit anti-TbPEX19- (middle panel) and mouse anti-GST antibodies (lower panel).
